# Ex Vivo Evaluation of Endothelial and Adventitial Temperature during Endovenous Laser Ablation Using a 1470-nm Radial 2ring Slim Fiber: Porcine Jugular Vein Model

**DOI:** 10.3400/avd.oa.26-00017

**Published:** 2026-09-25

**Authors:** Osamu Suzuki

**Affiliations:** Suzuki Medical Clinic, Suzuka, Mie, Japan

**Keywords:** endovenous laser ablation, 1470-nm radial 2-ring slim fiber, endothelial temperature, adventitial temperature, nerve injury

## Abstract

**Objectives:**

Endovenous laser ablation (EVLA) using a 1470-nm radial 2-ring slim fiber is widely performed, but optimal ablation settings remain empirical. This ex vivo study aimed to evaluate the effects of ablation conditions on endothelial and adventitial temperature dynamics, providing insights into both treatment efficacy and safety.

**Methods:**

Porcine jugular veins were used in an ex vivo model. EVLA was performed using a 1470-nm diode laser with the 2-ring slim fiber under 2 conditions: 5W with a linear endovenous energy density (LEED) of 25 J/cm and 6.5W with a LEED of 65 J/cm. Endothelial and adventitial temperatures were continuously measured at 0.1-second intervals. Cumulative durations with endothelial temperature ≥70°C and adventitial temperature ≥43°C were assessed.

**Results:**

Endothelial temperatures reached ≥70°C under both conditions, with a longer duration observed at 6.5W. Cumulative adventitial exposure ≥43°C was prolonged at 6.5W, whereas it remained minimal at 5W.

**Conclusions:**

In this ex vivo model, 6.5W with a LEED of 65 J/cm tended to produce more sustained endothelial and adventitial thermal exposure, whereas 5W with a LEED of 25 J/cm tended to produce lower adventitial exposure. Although further clinical validation is required, segment-specific settings may be reasonable.

## Introduction

Endovenous laser ablation (EVLA) has become a widely used treatment for varicose veins due to its minimally invasive nature and high occlusion rates. The introduction of 1470-nm diode lasers and radial slim fibers has enabled more uniform energy distribution across the vessel wall, reducing postoperative complications and expanding indications to tributary vein ablation. However, ablation settings are still largely based on empirical clinical experience, and the actual temperature dynamics within and outside the vessel wall remain insufficiently characterized. Postoperative nerve injury, in particular, is a complication that must be avoided, highlighting the need for basic data to scientifically guide safe ablation parameters. This study aimed to elucidate endothelial and adventitial temperature dynamics during ablation with a 1470-nm radial 2-ring slim fiber and, based on Moritz–Henriques thermal injury theory, to provide foundational insights for establishing rational and safe EVLA settings.^1)^

## Materials and Methods

The experimental model used in this study was developed with reference to the previous work by Proebstle et al.^2)^

### Specimens

Bilateral jugular vein specimens were provided as surplus tissues from animals used in an unrelated experiment. The specimens had luminal diameters of 4–8 mm and were derived from 2 female Landrace × Large White × Duroc (LWD) pigs, each approximately 2.5 months of age, weighing 37.04 and 40.24 kg, respectively. They were subsequently divided into multiple experimental specimens.

Specimens were prepared on the day before the experiment and stored in a refrigerated environment at 8°C while being kept moist with physiological saline. Approximately 3 hours before the experiment, the specimens were removed from refrigeration and allowed to equilibrate to room temperature (23°C) prior to use. Before the start of ablation, the specimen temperature was confirmed to have reached approximately 22°C–23°C.

After embedding the vein specimens in agar maintained at 22°C–23°C, temperature measurements were initiated within approximately 5–10 minutes to ensure thermal equilibration.

Because this study used ex vivo porcine jugular vein specimens obtained as surplus materials, no live animals were involved. Ethical considerations and institutional approval are described in the ethics approval section.

### Experimental model

Experiments were conducted in a laboratory maintained at 23°C. Agar (22°C–23°C) was placed in an acrylic chamber to simulate the perivascular environment after tumescent local anesthesia (TLA) injection. The vein specimens were embedded in the agar and lightly fixed under tension at both ends. This model aimed to reproduce the thermal buffering effect of TLA fluid on perivascular tissues (**Fig. 1D**).

**Fig. 1:**
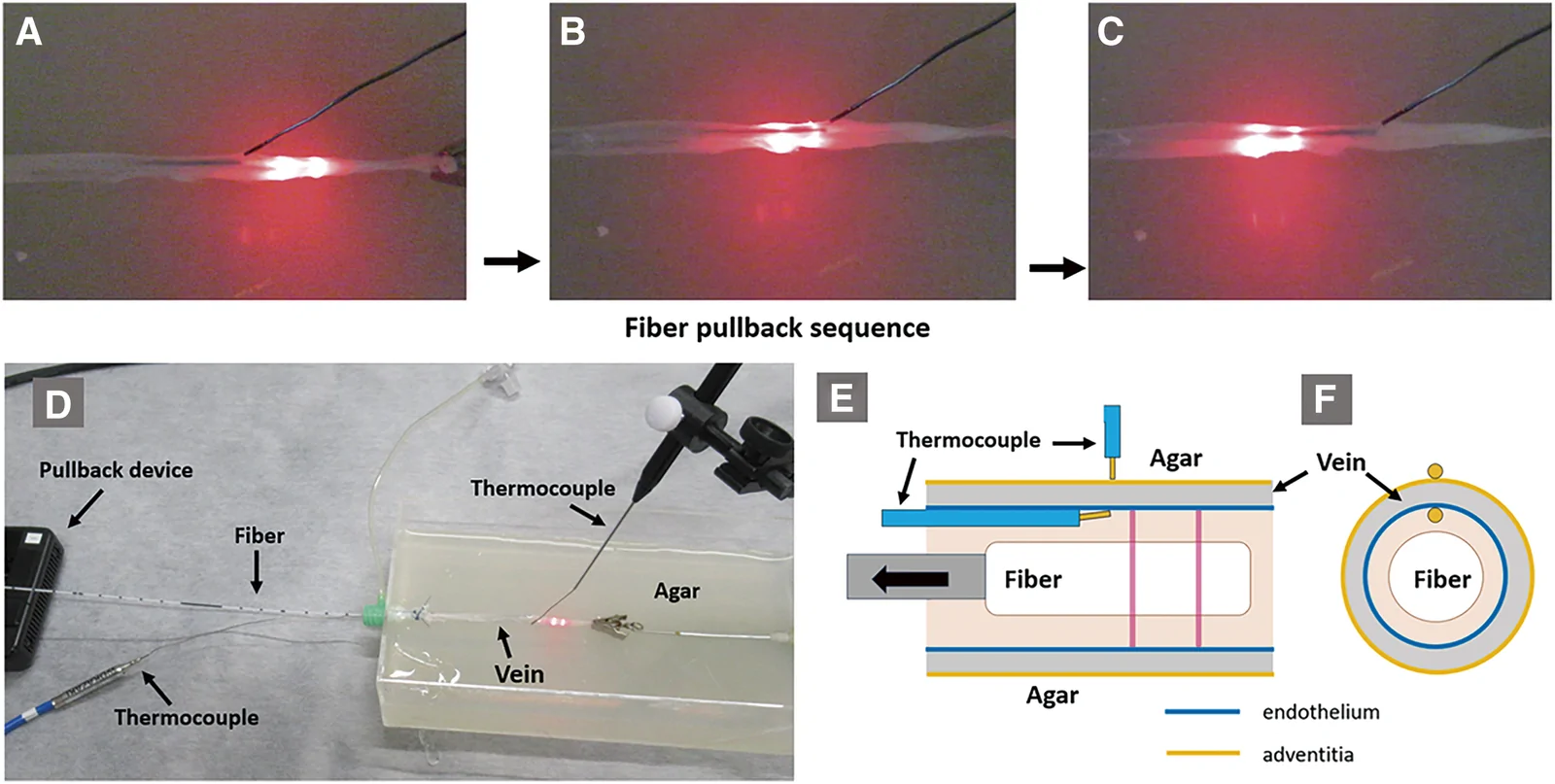
Experimental setup for measuring temperature changes during endovenous laser ablation. (**A**–**C**) Fiber pullback sequence. (**D**) Photograph of the ex vivo experimental setup demonstrating a laser fiber, thermocouples, a vein specimen, and the surrounding agar block simulating tumescent local anesthesia. (**E**) Longitudinal schematic illustration showing the positions of the 1470-nm radial 2-ring slim fiber and thermocouples placed in direct contact with the endothelial surface and on the adventitial surface of the vein wall. (**F**) Cross-sectional schematic illustration of the experimental configuration.

### Ablation and measurement equipment

EVLA was performed using a 1470-nm diode laser system (ELVeS; Biolitec, Jena, Germany) with an ELVeS Radial 2-ring Slim Fiber (CeramOptic, Bonn, Germany) (hereafter referred to as the 2-ring slim fiber).

Fiber pullback was controlled at a constant speed using an automated stage (SGSP20-85; Sigma Koki, Tokyo, Japan) (**Fig. 1A**–**1D**).

Temperature was measured using a 2-channel high-precision temperature/voltage unit (NR-TH08; KEYENCE, Osaka, Japan) and ultra-fine sheathed thermocouples (T35LC-200L2K9B; Okazaki Manufacturing, Hyogo, Japan). Temperature data were recorded continuously at 0.1-second intervals and analyzed via WAVE LOGGER X software (KEYENCE) and Microsoft Excel (Microsoft, Redmond, WA, USA).

### Experimental procedures and ablation settings

The 2-ring slim fiber connected to the laser generator was inserted into the lumen of each specimen and fixed to the pullback device. The tips of the thermocouples were positioned in direct contact with the endothelial surface and the adventitial surface, respectively, allowing simultaneous temperature measurement during ablation (**Fig. 1E** and **1F**).

Ablation was performed in continuous-wave mode. Based on clinically recommended settings, 2 ablation conditions were evaluated: 5W with a linear endovenous energy density (LEED) of 25 J/cm, recommended for distal segments of the saphenous trunk and tributary varicose veins, and 6.5W with a LEED of 65 J/cm, recommended for proximal saphenous trunk ablation (hereafter referred to as the 5W [LEED 25] and 6.5W [LEED 65] groups, respectively). Measurements were obtained from 5 specimens under the 5W condition and 8 specimens under the 6.5W condition.

Endothelial and adventitial temperatures were continuously recorded at 0.1-second intervals using a data logger and analyzed with WAVE LOGGER X software.

### Evaluation parameters

Temperature data were continuously recorded at 0.1-second intervals. For graphical presentation in **Figs. 2** and **3**, the 10 measurements obtained during each consecutive 1-second interval were first averaged separately for each specimen. The mean and standard deviation of these specimen-level 1-second averages were then calculated across the 5 specimens in the 5W (LEED 25) group and the 8 specimens in the 6.5W (LEED 65) group.

**Fig. 2:**
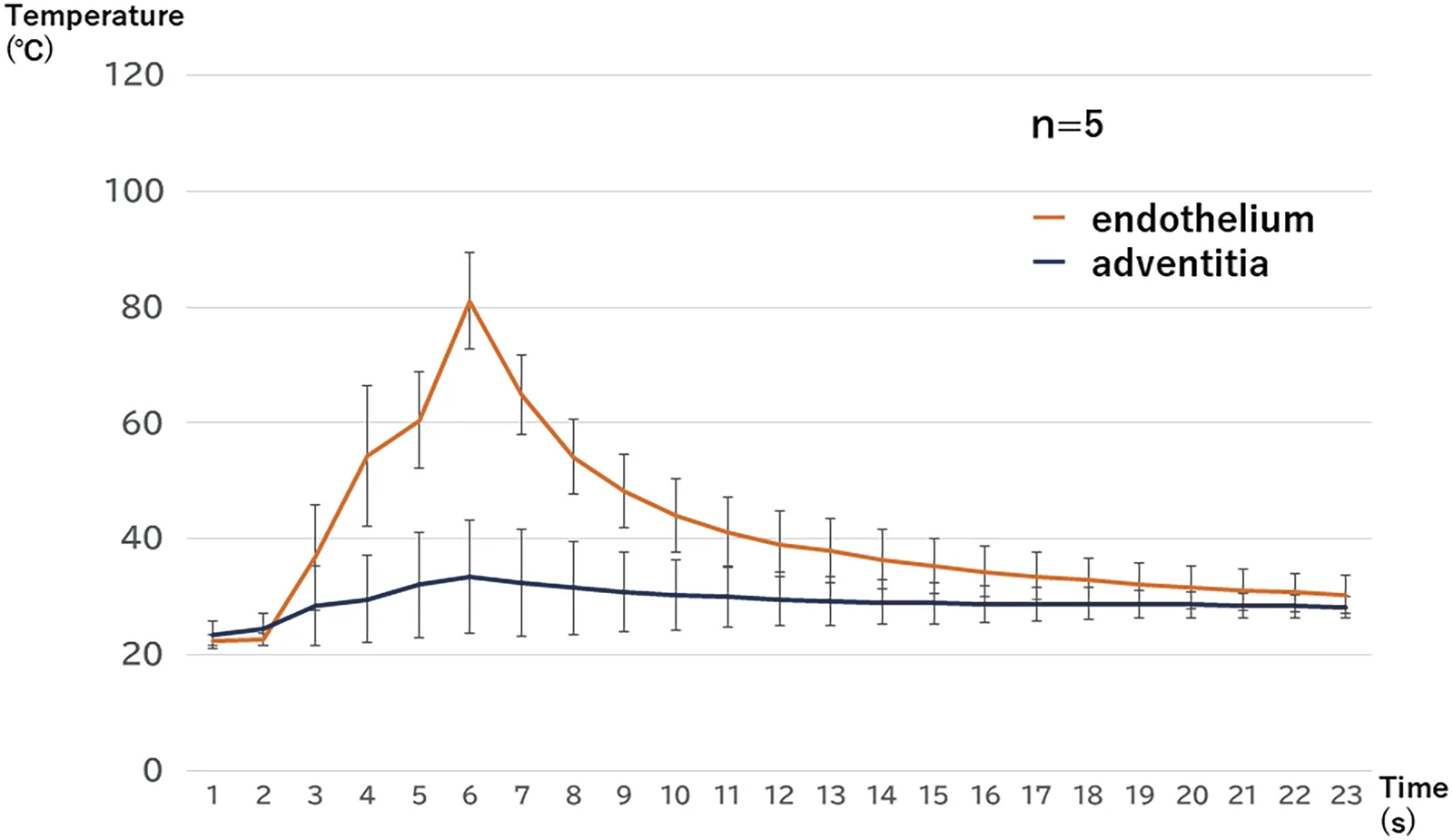
Endothelial and adventitial temperature–time curves at 5-W with a LEED of 25 J/cm. Endothelial and adventitial temperatures were continuously recorded at 0.1-second intervals during endovenous laser ablation performed at 5W with a linear endovenous energy density of 25 J/cm. For each specimen, the 10 measurements obtained during each consecutive 1-second interval were averaged. The graph shows the mean ± standard deviation of these specimen-level 1-second averages from 5 specimens. Because the plotted values are 1-second averaged group means, the maximum values of the curves do not necessarily correspond to the median specimen-level peak temperatures reported in **Tables 1** and **2**. LEED, linear endovenous energy density

**Fig. 3:**
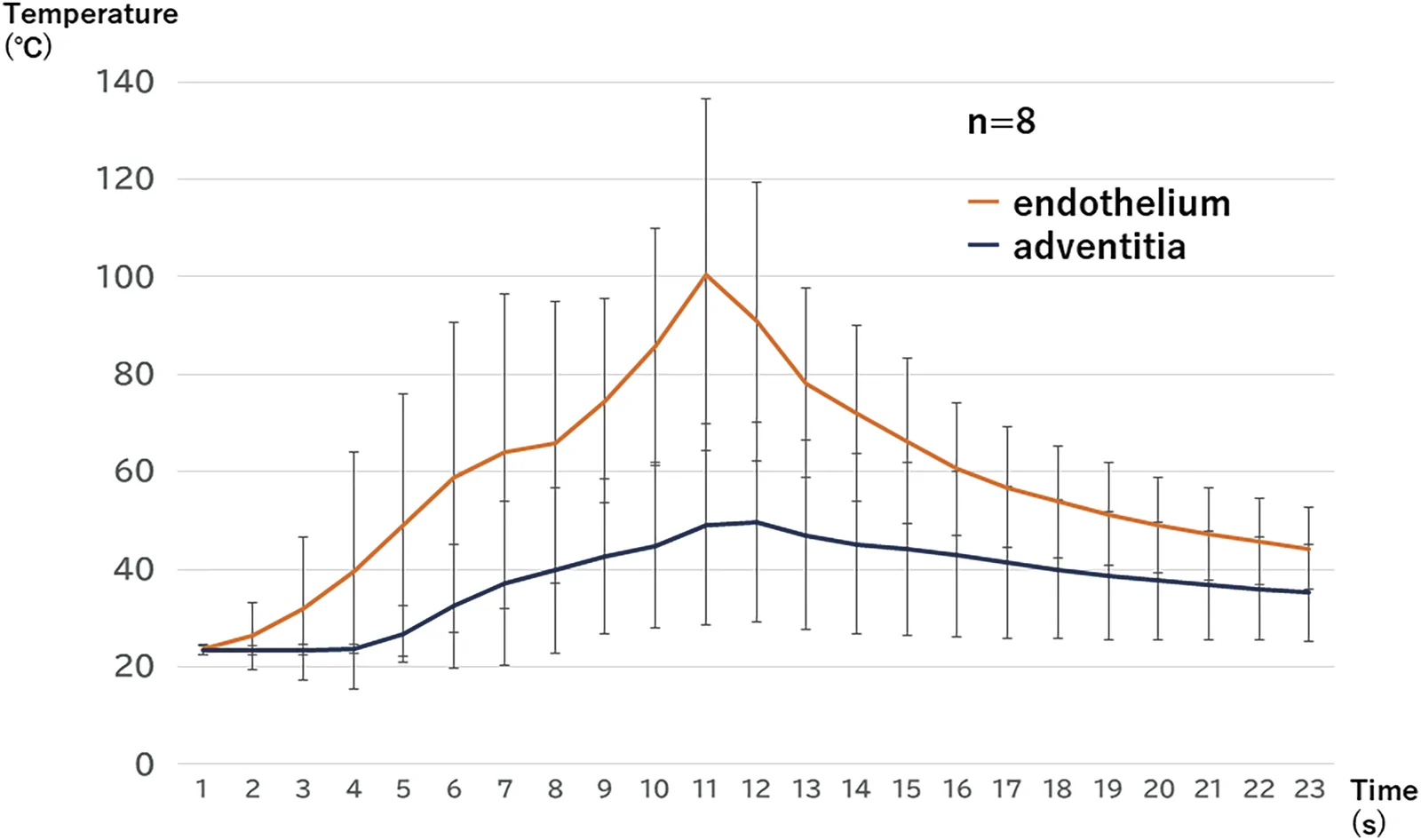
Endothelial and adventitial temperature–time curves at 6.5W with a LEED of 65 J/cm. Endothelial and adventitial temperatures were continuously recorded at 0.1-second intervals during endovenous laser ablation performed at 6.5W with a linear endovenous energy density of 65 J/cm. For each specimen, the 10 measurements obtained during each consecutive 1-second interval were averaged. The graph shows the mean ± standard deviation of these specimen-level 1-second averages from 8 specimens. Because the plotted values are 1-second averaged group means, the maximum values of the curves do not necessarily correspond to the median specimen-level peak temperatures reported in **Tables 1** and **2**. LEED, linear endovenous energy density

For each specimen, the peak endothelial and adventitial temperatures were defined as the highest temperatures recorded during the 0.1–23.0-second observation period using the original data sampled at 0.1-second intervals. Group values were calculated from the specimen-level peak temperatures and are presented as median [interquartile range].

Based on previous studies, the cumulative duration during which the endothelial temperature remained at or above 70°C, a threshold reported to induce endothelial and intimal injury sufficient for vein occlusion, was calculated for each specimen using the original 0.1-second interval data.^1)^

Similarly, the cumulative duration during which the adventitial temperature reached or exceeded 43°C, a threshold associated with an increased risk of thermal injury to nerves and other perivascular tissues, was calculated for each specimen using the original 0.1-second interval data.^2,3)^

For each specimen, the number of measurements meeting or exceeding the respective temperature threshold was multiplied by 0.1 seconds to obtain the cumulative exposure duration. Measurements exactly equal to the threshold were included, and specimens that did not reach the threshold were assigned a cumulative duration of 0 seconds. Group values are presented as median [interquartile range].

Using these parameters, endothelial and adventitial temperature profiles, peak temperatures, and cumulative exposure durations above the predefined temperature thresholds were evaluated for each ablation condition.

### Statistical analysis

Because of the small sample size and the potentially non-normal distribution of the data, intergroup comparisons were performed using a 2-sided exact permutation Mann–Whitney U test. Statistical analyses were conducted using a custom script written in Python version 3.13.5. For each comparison, all possible allocations of the 13 specimens into groups of 5 and 8 were enumerated. Tied observations were assigned average ranks. A P value <0.05 was considered statistically significant.

## Results

This study was conducted as an ex vivo experiment in which a vein model was embedded in agar gel simulating TLA at room temperature. Accordingly, the baseline values of both endothelial and adventitial temperatures presented in this section reflect the ambient temperature of the vein model within the agar gel and do not represent physiological body temperature.

The temperature curves shown in **Figs. 2** and **3** represent the mean ± standard deviation of specimen-level 1-second averages. Peak temperatures and cumulative exposure durations were calculated separately for each specimen from the original 0.1-second data and are summarized as median [interquartile range] in **Tables 1** and **2**.

**Table 1:** Endothelial peak temperature and cumulative duration at or above 70°C

Ablation setting	Peak endothelial temperature (°C)	P value	Cumulative duration ≥70°C (s)	P value
5W, LEED 25 J/cm	88.5 [79.3–97.7]	0.354	1.6 [1.1–2.3]	0.093
6.5W, LEED 65 J/cm	95.5 [85.0–116.4]		5.4 [3.6–11.8]	

Peak temperature was defined as the highest temperature recorded for each specimen during the 0.1–23.0-second observation period using the original data sampled at 0.1-second intervals. Cumulative duration was calculated as the number of measurements at or above 70°C multiplied by 0.1 seconds. Data are presented as median [interquartile range]. P values were calculated using a 2-sided exact permutation Mann–Whitney U test.

LEED, linear endovenous energy density

**Table 2:** Adventitial peak temperature and cumulative duration at or above 43°C

Ablation setting	Peak adventitial temperature (°C)	P value	Cumulative duration ≥43°C (s)	P value
5W, LEED 25 J/cm	30.1 [29.5–31.7]	0.222	0.0 [0.0–0.0]	0.217
6.5W, LEED 65 J/cm	52.3 [32.8–66.9]		4.4 [0.0–13.6]	

Peak temperature was defined as the highest temperature recorded for each specimen during the 0.1–23.0-second observation period using the original data sampled at 0.1-second intervals. Cumulative duration was calculated as the number of measurements at or above 43°C multiplied by 0.1 seconds; specimens that did not reach 43°C were assigned 0 seconds. Data are presented as median [interquartile range]. P values were calculated using a 2-sided exact permutation Mann–Whitney U test.

LEED, linear endovenous energy density

### Endothelial temperature changes

#### 5W (LEED 25) (n = 5)

The baseline endothelial temperature before ablation was 22.14°C ± 0.80°C, reflecting the temperature of the vein model within the agar gel simulating TLA rather than physiological body temperature.

The peak endothelial temperature was 88.5 [79.3–97.7]°C. The cumulative duration at or above 70°C was 1.6 [1.1–2.3] seconds (**Table 1**).

#### 6.5W (LEED 65) (n = 8)

The baseline endothelial temperature before ablation was 22.58°C ± 0.86°C, similar to that observed in the 5W (LEED 25) group.

The peak endothelial temperature was 95.5 [85.0–116.4]°C (**Table 1**).

The peak endothelial temperature did not differ significantly between the 5W (LEED 25) and 6.5W (LEED 65) groups (P = 0.354).

The cumulative duration at or above 70°C was 5.4 [3.6–11.8] seconds and tended to be longer than that in the 5W (LEED 25) group, although the difference did not reach statistical significance (P = 0.093) (**Table 1**).

### Adventitial temperature changes

#### 5W (LEED 25) (n = 5)

The baseline adventitial temperature before ablation was 22.00°C ± 0.60°C, reflecting the temperature of the vein model embedded in agar gel rather than physiological body temperature.

The peak adventitial temperature was 30.1 [29.5–31.7]°C. In most specimens, the adventitial temperature remained below 43°C (**Fig. 2** and **Table 2**).

Only 1 specimen (20%) reached an adventitial temperature of 43°C or higher. The cumulative duration at or above 43°C was 0.0 [0.0–0.0] seconds (**Table 2**).

#### 6.5W (LEED 65) (n = 8)

The baseline adventitial temperature before ablation was 23.01°C ± 1.01°C. The peak adventitial temperature was 52.3 [32.8–66.9]°C, and 4 specimens (50%) reached 43°C or higher (**Fig. 3** and **Table 2**).

The peak adventitial temperature did not differ significantly between the 5W (LEED 25) and 6.5W (LEED 65) groups (P = 0.222).

The cumulative duration at or above 43°C was 4.4 [0.0–13.6] seconds and tended to be longer than that in the 5W (LEED 25) group, although the difference did not reach statistical significance (P = 0.217) (**Table 2**).

## Discussion

Previous basic studies using ex vivo models have shown that the therapeutic effect of EVLA is primarily due to thermal injury to the vein wall, particularly the endothelium and the intima.^2)^ Furthermore, mathematical modeling analyses have suggested that thermal injury induced by EVLA should be evaluated not only by peak temperature but also by its cumulative effect over time, as represented by the product of temperature and exposure duration.^3)^

Comprehensive reviews of EVLA mechanisms indicate that the underlying physiological and thermal responses, including direct temperature measurements of the vein wall and adjacent tissues, are still not fully elucidated and remain incompletely documented.^4)^

To the best of our knowledge, no ex vivo experimental model using a 1470-nm radial 2-ring slim fiber under identical conditions has previously reported simultaneous direct measurements of endothelial and adventitial temperatures. Therefore, the present study provides novel experimental data that quantitatively evaluate both therapeutic efficacy and the risk of thermal injury during EVLA.

Peripheral nerve injury is a well-recognized adverse event following EVLA for varicose veins. In particular, several studies have reported a higher incidence of peripheral nerve injury when the great saphenous vein (GSV) is ablated to the distal below-knee segment.^5–7)^ Accordingly, the guidelines of the Japanese Society of Phlebology recommend that ablation of the below-knee GSV should generally be avoided whenever possible, with additional postoperative sclerotherapy considered when necessary.^7)^

In clinical practice, recommended ablation settings are established to achieve high occlusion rates of the saphenous trunk, and when a 1470-nm radial 2-ring slim fiber is used, an output power of 6.5W (LEED 65) represents one of the standard settings. However, applying the same ablation conditions used for the proximal segment to the below-knee GSV has been suggested to increase the risk of saphenous nerve injury.^8)^

In a clinical study investigating nerve injury after EVLA using a 1470-nm radial 2-ring slim fiber, Utoh et al. reported that when ablation was performed with a LEED of 59.6 J/cm, the incidence of nerve injury was 0.6% when the ablated length of the GSV was less than 40 cm, whereas it increased to 3.1% when the ablated length was 40 cm or longer. Furthermore, they demonstrated that reducing the LEED to 43 J/cm for below-knee ablation decreased the incidence to 1.7%, and that under low-LEED conditions of 20–25 J/cm, below-knee GSV occlusion could be achieved without causing saphenous nerve injury.^8–10)^

These reports indicate that optimizing ablation energy can balance treatment efficacy with the risk of nerve injury and have contributed to advances in clinical techniques for below-knee GSV ablation. Meanwhile, endovenous ablation for tributary varicose veins has been performed internationally for many years; in 2025, however, endovenous ablation using a 1470-nm radial 2-ring slim fiber was newly covered by national health insurance in Japan and was explicitly described as one of the standard treatment options in the guidelines of the Japanese Society of Phlebology. In these guidelines, the recommended ablation settings for tributary varicose veins are 5W with a LEED of 25–30 J/cm.^11)^

However, with respect to these ablation settings, evaluations based on the temperature dynamics of the endothelium and adventitia have not yet been sufficiently investigated.

The present study was designed to examine these clinically used ablation conditions from a fundamental perspective—namely, to quantitatively assess the “balance between treatment efficacy and safety” by ensuring endothelial temperatures sufficient for vein occlusion while maintaining adventitial temperatures below thresholds associated with nerve injury, from the viewpoints of temperature and exposure time.

As a classical investigation into thermal tissue injury, in 1947 Moritz AR and Henriques FC demonstrated that tissue damage is governed by the product of temperature and exposure time, thereby establishing the foundation of the so-called thermal dose theory. In their study, they reported that tissue injury can occur even after extremely short exposures at approximately 70°C, whereas the risk of tissue injury is extremely low at temperatures below 43°C.^1)^

Based on these findings, the present study calculated the cumulative duration during which the endothelial temperature was maintained at or above 70°C, a temperature threshold reported to induce endothelial and intimal injury sufficient for vein occlusion. In addition, for adventitial temperature, the cumulative duration during which the temperature reached or exceeded 43°C—a threshold associated with an increased risk of nerve and other tissue injury in the perivascular region—was calculated. Accordingly, the present study evaluated peak temperatures and cumulative exposure durations at or above the predefined temperature thresholds under each ablation condition.^2,3)^

With regard to the relationship between neural tissue and temperature, Haveman et al. showed that thermal exposure exceeding 44°C for more than 30 minutes may lead to irreversible nerve injury.^12)^ Furthermore, experimental studies in rats have reported that exposure at 47°C for 30 minutes results in reversible nerve injury, whereas exposure at 58°C for 10 minutes causes irreversible nerve injury.^13)^

All of these findings support the validity of the Moritz–Henriques theory and suggest that, in EVLA, evaluating endothelial and adventitial temperature changes from a temperature–time perspective may allow the identification of rational ablation conditions that balance treatment efficacy with safety. Based on this theoretical background, the present study selected 70°C as the temperature threshold for evaluating endothelial and intimal injury and 43°C as the threshold for assessing the risk of nerve injury.

In this study, ablation conditions with a high likelihood of future clinical application were defined as the 5W (LEED 25) group and the 6.5W (LEED 65) group. Endothelial and adventitial temperatures were measured under these conditions, and the relationship between temperature and exposure time was further analyzed to examine the circumstances under which each ablation condition could be considered rational in terms of both treatment efficacy and safety.

### Endothelial temperature and vein occlusion efficacy

The occlusive effect of EVLA is considered to result from irreversible thermal injury to the venous endothelium and media. According to the Moritz–Henriques theory, when endothelial temperature reaches ≥70°C, irreversible endothelial damage is likely to occur even with short exposure durations, leading to subsequent vein occlusion.

In the present study, endothelial temperatures reached ≥70°C in both the 5-W (LEED 25) and 6.5-W (LEED 65) groups. Both peak endothelial temperature and the cumulative duration during which temperature remained ≥70°C tended to be higher in the 6.5W (LEED 65) group; however, no statistically significant differences were observed between the groups.

Nevertheless, because the cumulative duration above 70°C was relatively longer in the 6.5W (LEED 65) group, this setting may be associated with more consistent thermal exposure, which could contribute to stable vein occlusion; however, this observation is based on a simplified ex vivo model and should not be interpreted as evidence of clinical durability.

In contrast, in smaller-diameter below-knee GSV segments and tributary varicose veins, the 5W (LEED 25) setting may provide endothelial thermal injury that could contribute to vein occlusion; however, the relatively short duration above 70°C raises the possibility of reduced occlusion durability, which requires further clinical validation. Therefore, these findings should be interpreted cautiously as preliminary observations derived from a simplified ex vivo model rather than as definitive evidence of clinical efficacy.

In addition, because several comparisons did not reach statistical significance, these findings should be interpreted as trends rather than definitive differences.

### Adventitial temperature and risk of nerve injury

Peripheral nerve injury is a well-recognized complication after EVLA and is considered to result primarily from thermal damage to nerves caused by heat conduction to the venous adventitia and surrounding tissues.

In the present study, the median peak adventitial temperature was 52.3°C in the 6.5-W (LEED 65) group and 30.1°C in the 5W (LEED 25) group. The median cumulative duration at or above 43°C was 4.4 seconds in the 6.5W (LEED 65) group and 0.0 seconds in the 5W (LEED 25) group. Both parameters tended to be higher in the 6.5W (LEED 65) group; however, the differences did not reach statistical significance.

These findings may have been influenced by the limited sample size and variability in measurement conditions, indicating the need for further investigation. Nevertheless, the consistently higher adventitial peak temperature and longer cumulative duration above 43°C observed in the 6.5W (LEED 65) group may be associated with a tendency toward higher adventitial thermal exposure. In contrast, the 5W (LEED 25) group showed lower adventitial temperatures and shorter exposure times above 43°C, suggesting a tendency toward lower adventitial thermal exposure.

However, these findings are based on indirect temperature measurements in an ex vivo model without direct nerve temperature assessment or histological validation. Furthermore, the duration of temperature elevation observed in this study was substantially shorter than that reported in previous studies of thermal nerve injury. Therefore, these results should not be interpreted as direct evidence of clinical nerve injury risk but rather as relative indicators for comparison between ablation settings.

Taken together, these results suggest that selective adjustment of ablation settings according to anatomical location may be a reasonable approach for balancing treatment efficacy and the risk of nerve injury. However, these findings are based on a simplified ex vivo model and should not be interpreted as definitive clinical recommendations.

### Thermal characteristics of the 2-ring fiber and the influence of ablation settings

The radial 2-ring slim fiber is designed to deliver laser energy circumferentially through 2 longitudinally separated prism structures, thereby producing sequential energy delivery to the venous wall during fiber pullback. This configuration is intended to distribute thermal energy relatively uniformly around the vessel circumference and has been reported to reduce fiber sticking and facilitate effective ablation even under relatively low linear endovenous energy density conditions.^14)^

Although the 2-ring structure results in sequential laser emission from 2 distinct positions, the temperature response measured at a single point on the venous wall represents the combined effects of direct laser energy absorption, heat conduction within the wall, cooling between successive exposures, and the timing of fiber passage relative to the thermocouple. Therefore, individual temperature peaks cannot necessarily be attributed reliably to either the first or second ring, particularly when the temperature elevations overlap or when the timing of the maximum temperature differs among specimens.

In the present study, the 5W (LEED 25) setting produced a shorter elevation of endothelial temperature and limited adventitial thermal exposure, whereas the 6.5W (LEED 65) setting was associated with a longer duration of elevated endothelial and adventitial temperatures. Although the between-group differences did not reach statistical significance, these findings suggest that ablation power and pullback speed influence not only the maximum temperature reached but also the duration of thermal exposure within and outside the venous wall.

Thermal tissue injury is determined not solely by peak temperature but by the combined effects of temperature and exposure duration, as described by thermal dose models such as the Arrhenius integral.^15)^ Accordingly, a relatively moderate temperature maintained for a longer period may produce substantial thermal injury, whereas a brief temperature elevation may result in a smaller cumulative thermal effect. From this perspective, the longer cumulative exposure observed with the 6.5W (LEED 65) setting may provide more sustained thermal effects on the venous wall, while also potentially increasing thermal exposure of the adventitia and adjacent tissues.

Conversely, the shorter adventitial exposure observed with the 5W (LEED 25) setting may be advantageous in anatomical regions where minimizing heat transfer to adjacent nerves is particularly important. These observations provide a physiological rationale for selecting ablation settings according to the treated venous segment rather than applying a single setting uniformly to all anatomical locations. However, because this study did not directly measure nerve temperature or histological injury, the findings should be interpreted as comparative thermal data rather than direct evidence of clinical efficacy or nerve injury risk.

### Clinical implications for the selection of ablation settings

In clinical practice, the selection of balanced ablation settings is critically important to ensure sufficient thermal energy for effective endothelial occlusion while maintaining adventitial temperatures below the threshold associated with nerve injury risk. The results of the present study indicate that the ablation setting of 6.5W (LEED 65), commonly used for truncal saphenous vein ablation, may be associated with more consistent endothelial thermal exposure; however, it also tends to prolong the duration of adventitial temperature exposure above 43°C. Accordingly, careful consideration of the anatomical relationship between the treated vein and adjacent nerves is required when applying this setting.

In contrast, the 5W (LEED 25) setting was associated with minimal elevation of adventitial temperature and a markedly shorter duration of high-temperature exposure, suggesting a tendency toward lower adventitial thermal exposure. This setting may therefore be considered a potentially reasonable approach in anatomical regions where nerve injury avoidance should be prioritized, such as the below-knee GSV and tributary varicose veins.

Taken together, the present findings suggest that a segment-specific approach to ablation settings may be reasonable: 6.5W (LEED 65) may be considered in situations where reliable occlusion is prioritized, whereas 5W (LEED 25) may be considered when reducing the risk of nerve injury is of particular importance. However, these findings should be regarded as preliminary and hypothesis-generating, because this study was performed in a simplified ex vivo model without blood flow, tissue perfusion, variable TLA distribution, or direct nerve measurements.

The Japanese Society of Phlebology guidelines report an incidence of nerve injury of 2.5% (range, 0%–9.1%) for tributary vein ablation; however, this figure is derived from pooled analyses of previous international studies that included heterogeneous ablation devices, energy settings, and procedural techniques.^11)^

In contrast, Utoh et al. reported single-center clinical outcomes using a 1470-nm radial 2-ring slim fiber under well-defined ablation conditions, demonstrating that the incidence of nerve injury varied substantially depending on the ablation length and LEED settings.^8–10)^

With the use of the insurance-approved 1470-nm radial 2-ring slim fiber and ablation performed using 5W (LEED 25) as a reference setting, the incidence of nerve injury may be further reduced; however, prospective clinical validation is warranted.

These findings should be interpreted within the limitations of a simplified ex vivo model and should not be directly extrapolated to clinical practice.

### Limitations

This study provides experimental insight into the widely accepted clinical observation that lowering ablation energy reduces the risk of nerve injury by examining endothelial and adventitial temperatures and the duration of high-temperature exposure. However, several limitations should be noted. First, the study used an ex vivo model, which does not fully reproduce blood flow, thermal dissipation in living tissues, or the precise anatomical course of peripheral nerves. A limitation of this study is that the baseline specimen and surrounding temperatures were approximately 22°C–23°C, which is lower than in vivo body temperature. Therefore, the absolute temperatures measured in this ex vivo model may underestimate those observed clinically. However, the relative differences in temperature profiles between treatment settings are likely to remain valid. Therefore, absolute temperature values should be interpreted with caution and not directly extrapolated to in vivo settings. Because venous wall thickness was not directly measured in this study, the influence of wall thickness on heat conduction from the endothelium to the adventitia could not be evaluated. Furthermore, a quantitative comparison of wall thickness between the porcine jugular veins used in this study and human saphenous veins was not possible. Therefore, the suitability of the porcine model with respect to venous wall thickness could not be fully verified. Although the porcine specimens were selected to provide relatively uniform biological characteristics and vein diameters within the range commonly encountered in clinical endovenous ablation, the potential influence of differences in venous wall thickness between porcine and human veins cannot be excluded.

Second, the analysis focused on endothelial and adventitial temperatures, while perivascular tissue temperatures near nerves were not directly measured. Third, the effects of additional TLA on reducing adventitial temperature and preventing nerve injury were not evaluated. Finally, interindividual variability in anatomy, vein diameter, and surrounding tissues may influence temperature responses, so findings from the 5W (LEED 25) and 6.5W (LEED 65) settings should be regarded as fundamental experimental insights rather than definitive clinical thresholds. Therefore, the findings of this study should not be directly extrapolated to clinical practice.

## Conclusion

Using a 1470-nm diode laser system with a radial 2-ring slim fiber, endothelial and adventitial temperatures were continuously measured in porcine jugular vein specimens under different ablation settings, and their thermal dynamics were quantitatively evaluated.

Both ablation conditions produced endothelial temperatures at or above 70°C, whereas the 6.5W (LEED 65) group showed a tendency toward longer endothelial and adventitial thermal exposure. None of the between-group differences reached statistical significance.

These findings suggest that, under the simplified conditions of this ex vivo model, 6.5W (LEED 65) may be associated with more sustained vein wall thermal exposure, whereas 5W (LEED 25) may be associated with lower adventitial thermal exposure. Therefore, a segment-specific approach may be considered reasonable. However, these findings should be interpreted as preliminary, and further in vivo and clinical studies are required before definitive conclusions regarding efficacy and safety can be made.
